# Surgically Resected Gall Bladder: Is Histopathology Needed for All?

**DOI:** 10.1155/2016/9319147

**Published:** 2016-03-30

**Authors:** Vikash Talreja, Aun Ali, Rabel Khawaja, Kiran Rani, Sunil Sadruddin Samnani, Farah Naz Farid

**Affiliations:** ^1^Department of Surgery, Jinnah Medical College Hospital, S. R-6, 7/A, Korangi Industrial Area, Karachi-74900, Pakistan; ^2^Department of Family & Community Medicine, College of Medicine, King Faisal University, Saudi Arabia; ^3^Department of Obstetrics and Gynecology, Jinnah Medical College Hospital, S. R-6, 7/A, Korangi Industrial Area, Karachi-74900, Pakistan; ^4^Department of Emergency, Jinnah Medical College Hospital, S. R-6, 7/A, Korangi Industrial Area, Karachi-74900, Pakistan; ^5^Mind and Brain Serviceline, Aga Khan University Hospital, P.O. Box 3500, Stadium Road, Karachi, Pakistan

## Abstract

*Background.* Laparoscopic cholecystectomy is considered to be gold standard for symptomatic gall stones. As a routine every specimen is sent for histopathological examination postoperatively. Incidentally finding gall bladder cancers in those specimens is around 0.5–1.1%. The aim of this study is to identify those preoperative and intraoperative factors in patients with incidental gall bladder cancer to reduce unnecessary work load on pathologist and cost of investigation particularly in a developing world.* Methods.* Retrospective records were analyzed from January 2005 to February 2015 in a surgical unit. Demographic data, preoperative imaging, peroperative findings, macroscopic appearance, and histopathological findings were noted. Gall bladder wall was considered to be thickened if ≥3 mm on preoperative imaging or surgeons comment (on operative findings) and histopathology report. AJCC TNM system was used to stage gall bladder cancer.* Results.* 973 patients underwent cholecystectomy for symptomatic gallstone disease. Gallbladder carcinoma was incidentally found in 11 cases. Macroscopic abnormalities of the gallbladder were found in all those 11 patients. In patients with a macroscopically normal gallbladder, there were no cases of gallbladder carcinoma.* Conclusion.* Preoperative and operative findings play a pivotal role in determining incidental chances of gall bladder malignancy.

## 1. Introduction

Most of the general surgery cases are of gallstone disease. It affects 15% of western world, having an annual incidence of 1 in 200 [1]. In Asian population its prevalence is around 3–5%. Four out of 100 patients with gallstones present with symptoms ranging from simple biliary colic to complications related to it. Laparoscopic cholecystectomy is now a recommended gold standard treatment for symptomatic gall stone patients [2].

As a routine standard practice it is made compulsory for practitioners to submit all gallbladders removed surgically to be sent for histopathology to exclude any gallbladder pathologies that can have significant impact on management of patients like gallbladder malignancies. One of the studies done by Royal College of Pathologist suggests that it is mandatory to submit all gall bladder specimens for histopathology as many significant pathologies can present with normal morphological appearance [3]. Incidentally finding malignancy in gallbladder specimen is around 0.5–1.1%, whereas gall stones were present in 74–92% of patients with gall bladder malignancies [4, 5].

It has been a point of discussion that patients with incidental histopathological finding of gall bladder malignancy have suspicious features on investigations and preoperative findings. So a routine histopathology may not be necessary in all cases [6, 7]. This study aims to identify those preoperative and intraoperative features in patients diagnosed with incidental gall bladder cancer on histopathology to reduce unnecessary work load on pathologist and cost of investigation particularly in developing world.

## 2. Methods

A retrospective review was done of all patients who underwent cholecystectomy with or without gallstone disease over a ten-year period from January 2005 till February 2015 in a single surgical unit. The hospital records of patients were retrieved and reviewed: demographic data, preoperative imaging, and preoperative findings. Patients with morphologically abnormal findings on preoperative imaging were excluded from the study. Macroscopic appearance and histopathological findings were also recorded. Gall bladder wall was considered to be thickened if ≥3 mm on preoperative imaging or surgeons comment (on operative findings) and histopathology report. Normal thickness of gall bladder wall is reported to be 1-2 mm. AJCC TNM system was used to stage gall bladder cancer. Data was entered and analyzed using SPSS 20.0.

## 3. Results

Nine hundred and seventy-three patients underwent cholecystectomy during the study time period. Most of them were females 70.29% (681). Records of 9 patients were either missing or showing gross pathological appearance in preoperative imaging suggesting or having suspicion of gall bladder malignancy so they were excluded from the study. Average age of patients was 41.30 ± 8.43 years (range 26–68 years). Common presenting symptoms were pain at upper abdomen followed by dyspepsia as shown in Table 1.

Nine hundred and sixty-four patient histopathological data were collected. Chronic cholecystitis was found to be more common and seen in 756 patients, Xanthogranulomatous cholecystitis in 12 cases, acute cholecystitis in 61 cases, cholesterosis in 117 cases, and metaplasia/adenoma/dysplasia were found in 7 patients. Incidentally gall bladder cancer was found in 11 (1.14%) patients as shown in Table 2. 79 (8.1%) patients had thickened gall bladder wall on preoperative imaging. 413 (42.8%) of patients had macroscopic abnormality like thickened gall bladder wall, mucosal defects, or ulcers or polypoid lesions.

Out of 11 cases diagnosed with incidentally having gall bladder carcinoma, 3 were males and 8 were female patients. Mean age of patients was 54.18 ± 8.95 (range 41–68 years). Thickened gall bladder was found in 6 (54.54%) of patients in preoperative imaging study. All patients underwent laparoscopic cholecystectomy but 4/11 converted to open because of dense adhesions or difficulty in defining Calot's triangle. Macroscopic abnormal appearance was found in all of these cases presented with nonspecific signs and symptoms. Nodularity/polypoid projections were present in 4 cases, whereas mucosal defect was presented in 3 cases out of 11. Nine patients have macroscopic appearance of thickened gall bladder wall. Details of each case are shown in Table 3.

Most of the cases were T1 and T2 on TNM staging. None of the patients with normal morphology and macroscopic appearance had gall bladder malignancy found.

## 4. Discussion

Cancer of gall bladder usually manifests itself in advance stages and carries a poor prognosis. It is most common malignancy of extra-hepatic biliary system [8]. Treatment of gall bladder malignancy depends on stage of disease with which patient presents. For tumors of stage Tis and T1a simple cholecystectomy is considered to be effective management. Management of T1b is controversial between simple and radical cholecystectomy. Advance tumors are managed by radical resection. There is no role for adjuvant therapy in cases of advance gall bladder cancers [9, 10].

All specimens are sent for histopathology after cholecystectomy was performed for gall stones diseases. Main rationale behind this approach is to found incidentally present carcinoma of gall bladder which accounts for being 0.5–1.1%. This includes patients whose preoperative workup and intraoperative findings were not conclusive of carcinoma gall bladder [4, 5]. Also chronic cholecystitis and early stages of gall bladder cancer manifest as thickened gall bladder wall so it is difficult to judge exact histopathology on the basis of wall thickness [11].

In our series of 973 cases only 11 patients were diagnosed with incidental gall bladder cancer. In all of these 11 patients there were gall bladder wall thickness and macroscopic features like nodularity, papillary growths, or ulcers. No malignancy was identified in those who had normal gall bladder imaging and macroscopic appearance.

One of the studies performed by Dix et al. found incidental gall bladder malignancy in 0.38% of patients. All these cases had abnormal macroscopic findings and three patients out of 5 had abnormal findings on preoperative imaging studies. The author recommends that more selective approach for sending histopathology would need to be evidence based [12]. Another study by Darmas et al. also concluded in favor of selective histopathology for cholecystectomy. It would not be missing any incidental carcinoma; indeed it would be cost effective and time consuming for histopathologist [6].

Gross abnormalities of gall bladder cancers can be grouped into infiltrative, papillary, nodular, or mixed forms. Infiltrative form usually manifests itself as thick walled gall bladder while polypoid lesions were seen in cases of papillary projections. Nodular form usually presents as circumcised masses. Most common form of presentation is infiltrative pattern followed by papillary. Thus, by finding any of these macroscopic appearances one could get suspicion of likelihood of gall bladder cancer [13, 14].

There was a concern between dysplasia and early stages of gall bladder cancer, but treatment for both these conditions is simple cholecystectomy. Most of the patients in this group would benefit from simple cholecystectomy and radical procedures would not be needed [9, 10].

There had been conditions like Xanthogranulomatous cholecystitis and Mirizzi syndrome which can be associated with increased risk for gall bladder cancer. Rao et al. reported 6% of patients with gall bladder malignancy and Kwon and Sakaida reported it to be 10% [14]. Histopathological examination should be done in such cases where intraoperative and gross findings are suggestive of these conditions [15].

Studies have suggested that there had been about 40% of unnecessary investigations carried out in the absence of gross or macroscopic abnormalities. Routine histopathology is not only adding cost to patients care but also increasing load on pathology staff [16]. Average cost of processing specimen in Pakistan varies across hospital but average is around 20–40 USD (1 USD = 100) and average time spent by pathologist for one specimen is around 50–60 minutes including reporting of findings. In our study, 934 patients had chronic cholecystitis, acute cholecystitis, and cholesterolosis on examination and if their surgically resected specimen would not be sent for histopathological examination, then it would have save around 18000-37000 USD. Also load on pathologist would be reduced and it could save their 56000 minutes.

Major limitations of our studies were retrospective nature of study. A prospective study would signify implication of selective histopathology for surgically resected gall bladder for gall stone diseases.

## 5. Conclusion

Preoperative and operative findings play a pivotal role in determining incidental chances of gall bladder malignancy. A more selective approach based on preoperative imaging and macroscopic appearance would not only reduce cost but also reduce pathologist workload.

## Figures and Tables

**Table 1:** Common presenting symptoms.

Symptoms	Number of patients	Percentage%
Pain at upper abdomen	719	74.58
Intolerance to food (fat diet)	157	16.28
Nausea/vomiting	27	2.80
Palpable and tender gall bladder	61	6.32

**Table 2:** Histopathological findings of resected gall bladder specimen.

Diagnosis	Number of patients	Percentage%
Chronic cholecystitis	756	78.42
Acute cholecystitis	61	6.32
Cholesterolosis	117	12.13
Xanthogranulomatous cholecystitis	12	1.24
Metaplasia/adenoma/dysplasia	07	0.73
Carcinoma	11	1.14

**Table 3:** Brief description of patients with incidentally identified gall bladder carcinoma.

S. number	Age	Gender	Imaging findings	Surgery	Macroscopic appearance	Pathology	TNM stage
1	41	Female	Normal	Laparoscopic	Thickened gall bladder (4 mm)	Well differentiated	T1a
2	62	Male	Thickened wall (4.5 mm)	Laparoscopic to open	Thickened gall bladder, polypoid (6 mm)	Moderately differentiated	T2
3	59	Female	Normal	Laparoscopic	Thickened gall bladder, polypoid (4.5 mm)	Moderately differentiated	T2
4	53	Female	Thickened wall (3.4 mm)	Laparoscopic to open	Thickened gall bladder (5 mm)	Well differentiated	T1a
5	57	Female	Thickened wall (4 mm)	Laparoscopic	Thickened gall bladder, polypoid (7 mm)	Moderately differentiated	T2
6	48	Male	Normal	Laparoscopic	Mucosal ulcer (5 mm)	Carcinoma in situ	CIS
7	66	Female	Thickened wall (3.9 mm)	Laparoscopic to open	Thickened gall bladder (7 mm)	Well differentiated	T1b
8	68	Female	Thickened wall (4.1 mm)	Laparoscopic	Thickened gall bladder (6 mm)	Moderately differentiated	T2
9	51	Male	Thickened wall (3.8 mm)	Laparoscopic	Thickened gall bladder, mucosal ulcer, polypoid (8 mm)	Carcinoma in situ	T2
10	44	Female	Normal	Laparoscopic	Thickened gall bladder (6 mm)	Well differentiated	T1b
11	47	Female	Normal	Laparoscopic to open	Mucosal ulcer (5 mm)	Moderately differentiated	T2
